# Supplementary material for: Genomic epidemiology of SARS-CoV-2 reveals multiple lineages and early spread of SARS-CoV-2 infections in Lombardy, Italy
Source: Nat Commun. 2021 Jan 19;12:434. doi: 10.1038/s41467-020-20688-x (PMC7815831; doi:10.1038/s41467-020-20688-x)
Supplement: Supplementary file 1 — Supplementary Information [file 41467_2020_20688_MOESM1_ESM.pdf]

Supplementary Information

Title: Genomic epidemiology of SARS-CoV-2 reveals multiple lineages and early spread of SARS-CoV-2 infections in Lombardy, Italy

Running head: Genomic epidemiology of SARS-CoV-2 in Lombardy, Italy

Claudia Alteri[1§], Valeria Cento[1§], Antonio Piralla[2§], Valentino Costabile[3], Monica Tallarita[2], Luna Colagrossi[4], Silvia Renica[1], Federica Giardina[2], Federica Novazzi[2], Stefano Gaiarsa[2], Elisa Matarazzo[5], Maria Antonello[1], Chiara Vismara[6], Roberto Fumagalli[7], Oscar Massimiliano Epis[8], Massimo Puoti[9], Carlo Federico Perno[1,4✉], Fausto Baldanti[2,10]

[1]Department of Oncology and Hemato-oncology, University of Milan, Milan, Italy

[2]Molecular Virology Unit, Microbiology and Virology Department Fondazione IRCCS Policlinico San Matteo, Pavia, Italy

[3]Department of Pathophysiology and Transplantation, University of Milan, Milan, Italy

[4]Microbiology and Diagnostic Immunology, IRCCS, Bambino Gesù Children's Hospital, Rome, Italy

[5]Residency in Microbiology and Virology, University of Milan, Milan, Italy

[6]Chemico-clinical and Microbiological Analyses, ASST Grande Ospedale Metropolitano Niguarda, Milan, Italy

[7]Department of Anesthesiology, Critical Care and Pain Medicine, ASST Grande Ospedale Metropolitano Niguarda, 20162, Milan, Italy

[8]Rheumatology Unit, ASST Grande Ospedale Metropolitano Niguarda, 20162, Milan, Italy

[9]Infectious Diseases, ASST Grande Ospedale Metropolitano Niguarda, Milan, Italy

[10]Department of Clinical, Surgical, Diagnostic and Paediatric Sciences, University of Pavia, Pavia, Italy

[§] These authors contributed equally: CA, VCe, AP

[✉]email: cf.perno@uniroma2.it

Supplementary Results

Sampling criteria and patients' characteristics

For 9,251 patients we were able to retrieve information regarding sex, age, and residence. In order to exclude sampling bias that could affect viral diversity, only one patient per family unit was selected (n=7,617). In order to have the measure of viral load of the selected samples, samples with Ct available were retrieved (n=1,561 samples). To warrant high quality sequences and good genomic coverage, samples with Ct values >35 cycles (n=418) were excluded. Out of the remaining 1,143 patients, 371 samples were selected for inclusion, according to the geographical distribution of COVID-19 cases. In Supplementary Table 1, the characteristics of the 9,251 Sars-CoV-2 infected patients with sex, age, and residence information available, were compared with the 371 selected samples. Likelihood Ratio Test, followed by a multinomial logistic regression model to estimate 95% confidence intervals of odds ratios, was used to compare demographic and clinical findings between general and selected SARS-CoV-2 infected populations. By looking at sex, age distribution, the selected population is well representative of SARS-CoV-2 infected general population at that time (at the time of writing the epidemiology is substantially different). Prevalence of chronic comorbidities is also similar, with the exception of a higher prevalence of cardiovascular and lung diseases in the selected population, compared to the general population (33.2% vs. 24.5%, P<0.001 by Likelihood Ratio Test; and 14.2% vs. 11.3%, P=0.04 by Likelihood Ratio Test, respectively). Disease severity and evidence of interstitial pneumonia were largely comparable, even though a lower prevalence of critical COVID-19 cases was observed in the selected population (4.3% vs. 9.6% in the general population; P=0.001 by Likelihood Ratio Test). The most frequent symptom observed is fever in both populations (66.0% and 63.4%; P=0.290 by Likelihood Ratio Test), followed by cough and dyspnea, whose prevalence were lower in the selected population (46.0% vs. 52.2% in general population, P=0.001 by Likelihood Ratio Test; and 38.8% vs. 50.1% in general population, P<0.001 by Likelihood Ratio Test). The geographical distribution is also comparable between general and selected populations, with the exception of Milan, Pavia and Como. In this respect, it should be noted that this retrospective observational study involved two major hospitals localized in Milan and Pavia. Consequently, most of the SARS-

CoV-2 infected population resided in these two provinces (Milan: 31.1%; Pavia: 25.8%). In order to balance the geographical distribution in relation to population density and general prevalence of COVID-19 cases, patients from Milan and Pavia were under-sampled down to 20.6% and 19.2% of the selected population, respectively. A higher prevalence of patients residing in Como remained in the selected population in relation to the general population (19.2% vs. 8.7%, $P<0.001$ by Likelihood Ratio Test).

Overall, the study population was well representative of the whole Lombardy region, with the exception of the eastern part and northern valleys (i.e. Brescia, Mantua, Valtellina and Valcamonica, Figure 1).

Supplementary Methods

Phylogenetic analysis

Homoplasies checking

To account for regions which might potentially be the result of hypervariability or sequencing artifacts, alignment positions showing significant homoplasy were identified by a combined approach. Homoplasies were firstly identified using HomoplasyFinder, and then confirmed by Treetime (homoplasy setting).[1,2] In detail, MPBoot was run on the alignment to reconstruct the Maximum Parsimony tree and to assess branch support following 1000 replicates (−bb 1000). The resulting Maximum Parsimony treefile was used, together with the input alignment, to rapidly identify homoplasies using HomoplasyFinder.[1] To obtain a set of high confidence homoplasies, we then confirmed the results obtained by HomoplasyFinder using Treetime (homoplasy setting).[2] The top-10 significant homoplastic positions identified by TreeTime and confirmed in HomoplasyFinder were masked in the final alignment.

SARS-CoV-2 genome data set

In order to represent the global diversity of the lineages by the end of April 2020 while minimizing the impact of sampling bias, 395 GISAID deposited sequences were added to the 346 consensus sequences obtained by our samples.

The 395 GISAID sequences were selected as follows. All available whole-genome SARS-CoV-2 sequences (n=3244) on GISAID (gisaid.org) on 3 May 2020 were downloaded and submitted to the Pangolin application. Sequences from GISAID that were error-rich, and identical sequences from each country outbreak were removed. The exact date of virus collection was available for all sequences except for one genome from Lithuania for which only the month of viral collection (February, 2020) was available. In this case, the lack of tip date precision was accommodated by sampling uniformly across a 30-day window. Finally, the dataset was reduced to 395 sequences by only retaining the earliest, and the most recently sampled sequences from each country outbreak (range of dates: 2019, December 24 – 2020, April 4). Sequences were aligned using ClustalX and manually inspected in Bioedit. The final alignment was composed of 741 sequences 29,159 nucleotides long.

Maximum likelihood tree and Bayesian interference

In order to explore the phylogenetic structure of SARS-CoV-2, we used both the maximum likelihood (ML) and Bayesian coalescent methods. The ML phylogeny was estimated with IqTree[3] using the best-fit model of nucleotide substitution GTR+I.[4] Tree topology was assessed with the fast bootstrapping function with 1000 replicates. The ML tree was inspected in TempEst,[5] in order to define the correlation between genetic diversity (root-to-tip divergence) and time of sample collection (Supplementary Fig. 3). In order to obtain a corresponding time-scaled maximum clade credibility tree, a Bayesian coalescent tree analysis was undertaken with BEAST v1.10.5,[6] using the GTR+I substitution model with an exponential population growth tree prior and strict molecular clock, under a noninformative continuous-time Markov chain (CTMC) reference prior.[7] Taxon sets were defined and used to estimate the posterior probability of monophyly and the posterior distribution of the tMRCA of observed phylogenetic clusters. Four independent chains were run for 50 million states and parameters and trees were sampled every 1,000 states. Upon completion, chains were combined using LogCombiner after removing 10% of states as burn-in and convergence was assessed with Tracer (ESS>100). Monophyly and tMRCA (time to the most recent common ancestor) statistics were calculated for each taxon set from the posterior tree distribution.

The information regarding location and recent travel history of the most informative sequences for virus spread and clustering identified in the first Bayesian tree were incorporated in a second Bayesian tree interference,[8] in order to yield more robust reconstructions of virus spread. For genomes from patients with a recent travel history, the travel locations in the ancestral location reconstructions were used. A GTR+I substitution model with an exponential population growth tree prior and strict molecular clock, under a noninformative continuous-time Markov chain (CTMC) reference prior[7] was used. Two independent chains were run for 25 million states and parameters and trees were sampled every 1,000 states. Upon completion, chains were combined using LogCombiner after removing 10% of states as burn-in and convergence was assessed with Tracer (ESS>100).

The maximum clade credibility (MCC) trees were inferred from the Bayesian posterior tree distribution using TreeAnnotator and visualized with FigTree 1.4.4.[9]

Supplementary Table 1. Demographic, and clinical findings of general SARS-CoV-2 infected population and the 371 originally selected SARS-CoV-2 infected patients.

	General COVID-19 affected population, N=9,251	Sampled population for SARS-CoV-2 sequencing, N=371	Odds Ratio (Confidence Interval)[§]	P-value[§]
Demographics and clinical characteristics				
Age, years	72 (55-83)	72 (54-84)	0.99 (0.99-1.00)	0.736
18-39	684 (7.4)	34 (9.2)	1.15 (0.76-1.73)	0.173
40-49	906 (9.8)	37 (10.0)	1.03 (0.71-1.51)	0.878
50-59	1355 (14.6)	53 (14.4)	0.92 (0.65-1.29)	0.875
60-69	1344 (14.5)	48 (13.0)	0.89 (0.63-1.25)	0.398
70-79	1788 (19.3)	66 (17.9)	0.93 (0.68-1.26)	0.474
≥80	3174 (34.3)	131 (35.5)	1.04 (0.83-1.31)	0.623
Sex, Male	4923 (53.2)	207 (56.1)	1.13 (0.91-1.39)	0.258
Residency				
Milan	2867 (31.1)	76 (20.6)	0.51 (0.30-0.88)	<0.001
Como	803 (8.7)	71 (19.2)	2.65 (2.03-3.47)	<0.001
Pavia	2390 (25.8)	71 (19.2)	0.50 (0.29-0.87)	<0.001
Bergamo	790 (8.5)	37 (10.0)	1.20 (0.85-1.70)	0.297
Lecco	946 (10.2)	32 (8.7)	0.63 (034-1.16)	0.315
Lodi	589 (6.4)	34 (9.2)	1.14 (0.62-2.08)	0.669
Cremona	520 (5.6)	29 (7.9)	1.01 (0.54-1.90)	0.115
Other[a]	346 (3.7)	19 (5.1)	1.35 (0.82-2.23)	0.231
Chronic comorbidities[b]	285 (51.3)	162 (51.3)	1.00 (0.71-1.40)	0.918
Hypertension	186 (33.5)	114 (36.1)	1.13 (0.92-1.88)	0.133
Obesity	41 (7.4)	25 (7.9)	1.17 (0.60-2.26)	0.725
Diabetes	52 (9.4)	33 (10.4)	1.34 (0.74-2.44)	0.273
Cardiovascular disease	136 (24.5)	105 (33.2)	3.40 (2.16-5.34)	<0.001
Chronic obstructive lung disease	63 (11.3)	45 (14.2)	2.05 (1.15-3.64)	0.040
Malignancies	57 (10.3)	35 (11.1)	1.31 (0.74-2.29)	0.558
Chronic kidney disease	40 (7.2)	24 (7.6)	1.12 (0.58-2.20)	0.623
Chronic liver disease	13 (2.3)	4 (1.3)	0.33 (0.10-1.08)	0.061
Other[c]	37 (6.7)	28 (8.9)	2.5 (1.18-5.51)	0.017
Symptoms at admission[d]				
Fever	479 (63.4)	165 (66.0)	1.19 (0.86-1.63)	0.290
Cough	410 (54.2)	115 (46.0)	0.59 (0.44-0.81)	0.001
Dyspnea	379 (50.1)	97 (38.8)	0.52 (0.38-0.72)	<0.001
Time from symptoms-onset to SARS-CoV-2 diagnosis, weeks	0.48 (0.26-0.78)	0.29 (0.14-0.57)	0.09 (0.05-0.16)	<0.001
Disease severity[e]				
Mild	352 (46.3)	129 (50.8)	1.31 (0.97-1.77)	0.080
Moderate	163 (21.4)	57 (22.4)	1.09 (0.75-1.58)	0.636
Severe	172 (22.6)	57 (22.4)	0.98 (0.69-1.41)	0.929
Critical	73 (9.6)	11 (4.3)	0.32 (0.16-0.63)	0.001
Evidence of Interstitial Pneumonia[f]	410 (53.9)	126 (49.6)	0.77 (0.57-1.04)	0.089
SARS-CoV-2 rtPCR				
Mean cycle thresholds[g]	23.9 (19.6-29.3)	18.9 (16.9-20.1)	0.69 (0.66-0.72)	<0.001

Data are expressed as median (IQR), or N (%). [§]For comparisons of demographic and clinical findings between general and selected SARS-CoV-2 infected populations, a Likelihood Ratio Test followed by a multinomial logistic regression model to estimate 95% confidence intervals of odds ratios was used. Two-sided P-values are reported. [a]Other includes Brescia, Mantua, Monza and Brianza, Sondrio and Varese. [b]Data available for 556 patients. [c]Including: Crohn's disease (n=1), Hashimoto's thyroiditis (n=5), familial lipid disorders (n=10), rheumatoid arthritis (n=3), Amyotrophic Lateral Sclerosis (n=1), Parkinson' s disease (n=1), cognitive disorders (n=10), immunological disorders (n=5), Tuberculosis (n=1). [d]Data available for 756 patients. [e]Data available for 760 patients. [f]Diagnosed by X Ray or CT Scan. Data available for 5,578 patients. [g]Real-time reverse transcription PCR Ct (cycle threshold) values of these samples ranged from 9 to 35 (GeneFinderTM COVID-19 Plus RealAmp Kit, ELITech; AllplexTM 2019-nCoV Assay, Seegene; Corman VM, Landt O, Kaiser M, et al. Detection of 2019 novel coronavirus (2019-nCoV) by real-time RT-PCR. Euro Surveill. 2020;25(3):2000045. doi:10.2807/1560-7917.ES.2020.25.3.2000045; https://www.who.int/docs/default-source/coronaviruse/protocol-v2-1.pdf).

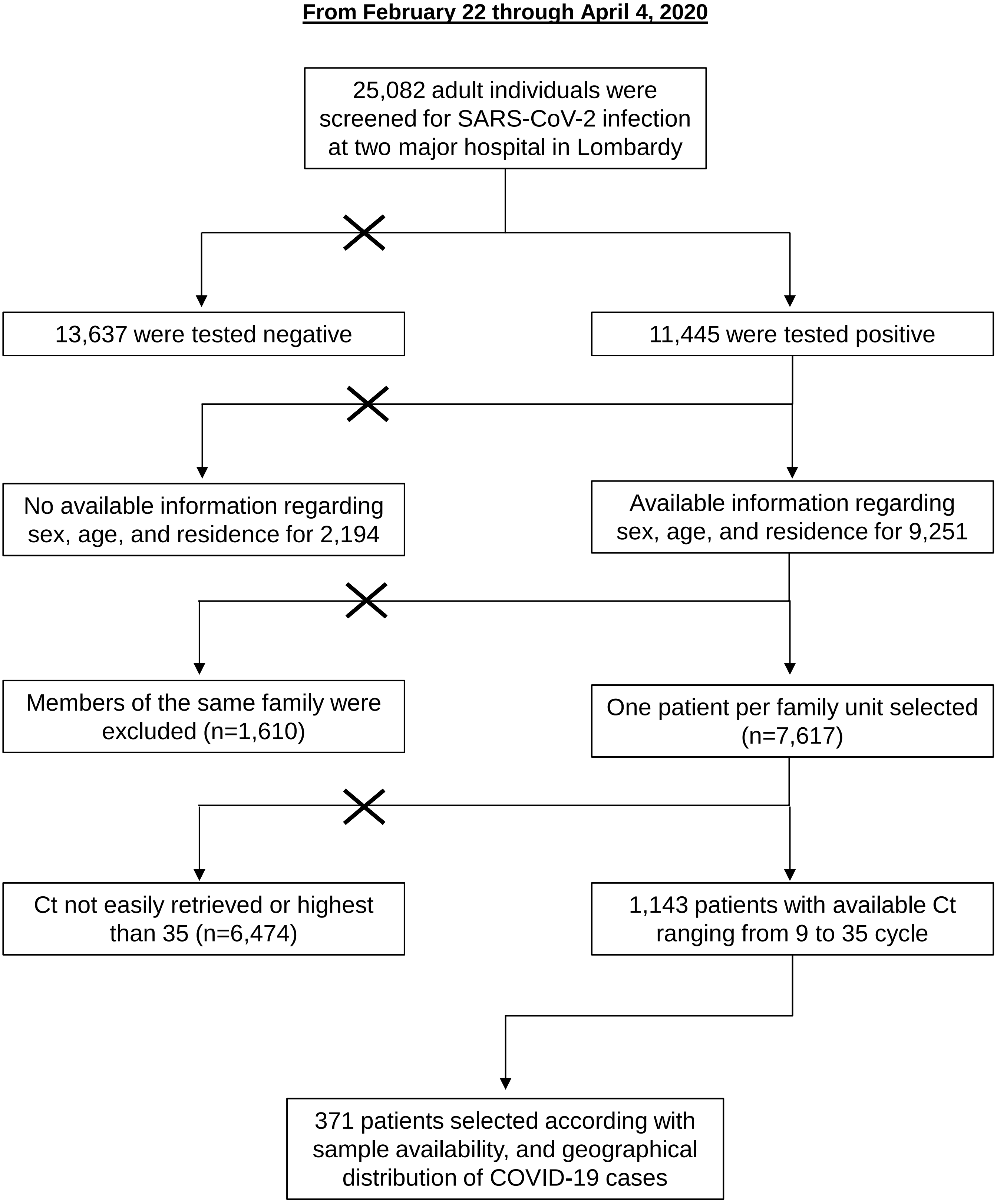

Supplementary Fig. 1. **Selection criteria for the 371 swab samples originally included in the study**

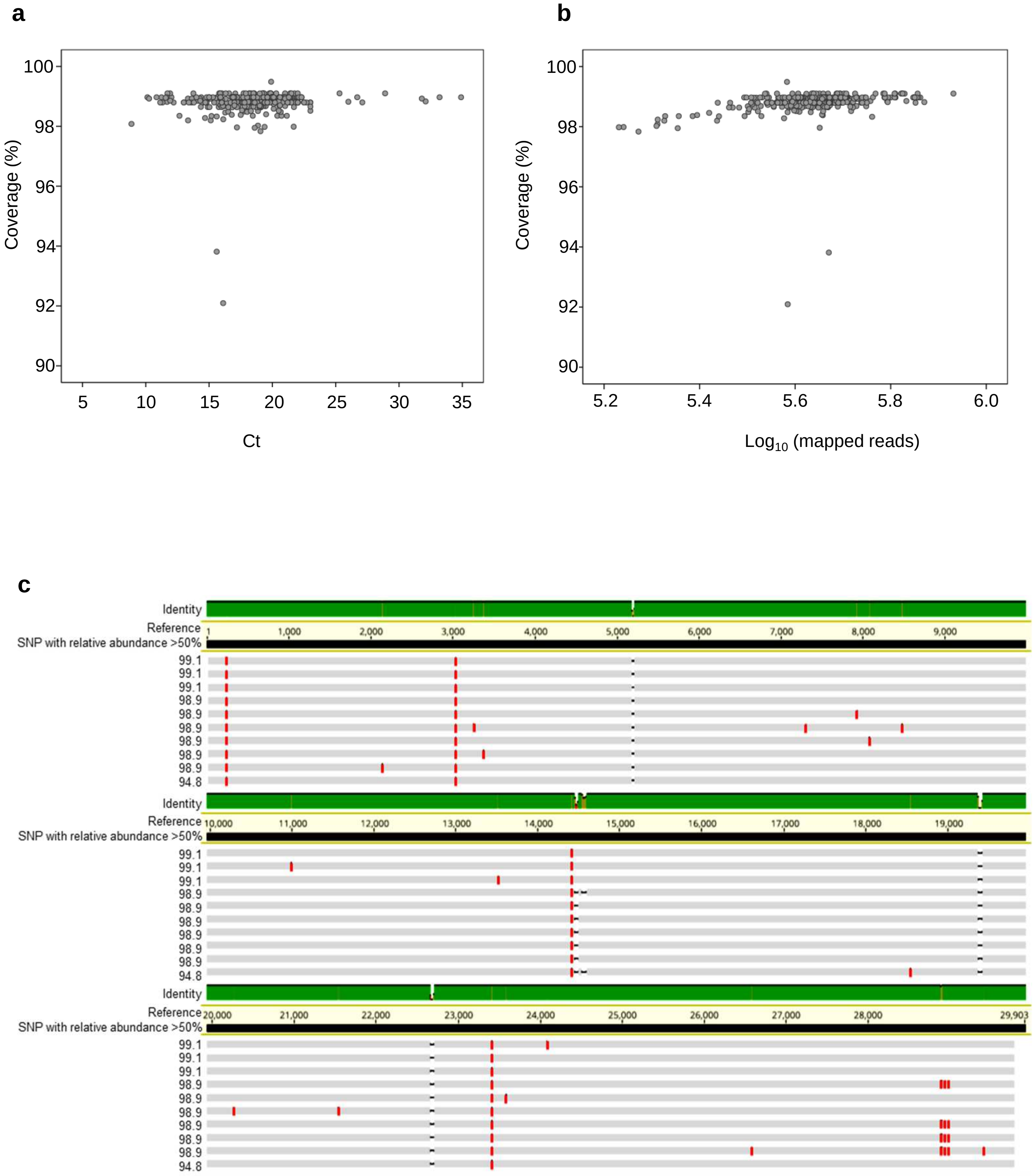

Supplementary Fig. 2. **Profile of SARS-CoV-2 Genome Sequences from Lombardy, Italy.** Plots of SARS-CoV-2 genome coverage against **a** rtPCR Ct Value and **b** the Number of Mapped Reads for the 355 samples described. Each sequence is represented by a dot. **c** Genome coverage map of the 10 SARS-CoV-2 samples representative of the genome coverage obtained. Single nucleotide polymorphisms (with respect to the reference genome NC_045512.2) are in red, and uncovered portions are indicated by black gaps.

rtPCR: real-time polymerase chain reaction; Ct: cycle threshold; SNP: single nucleotide polymorphism

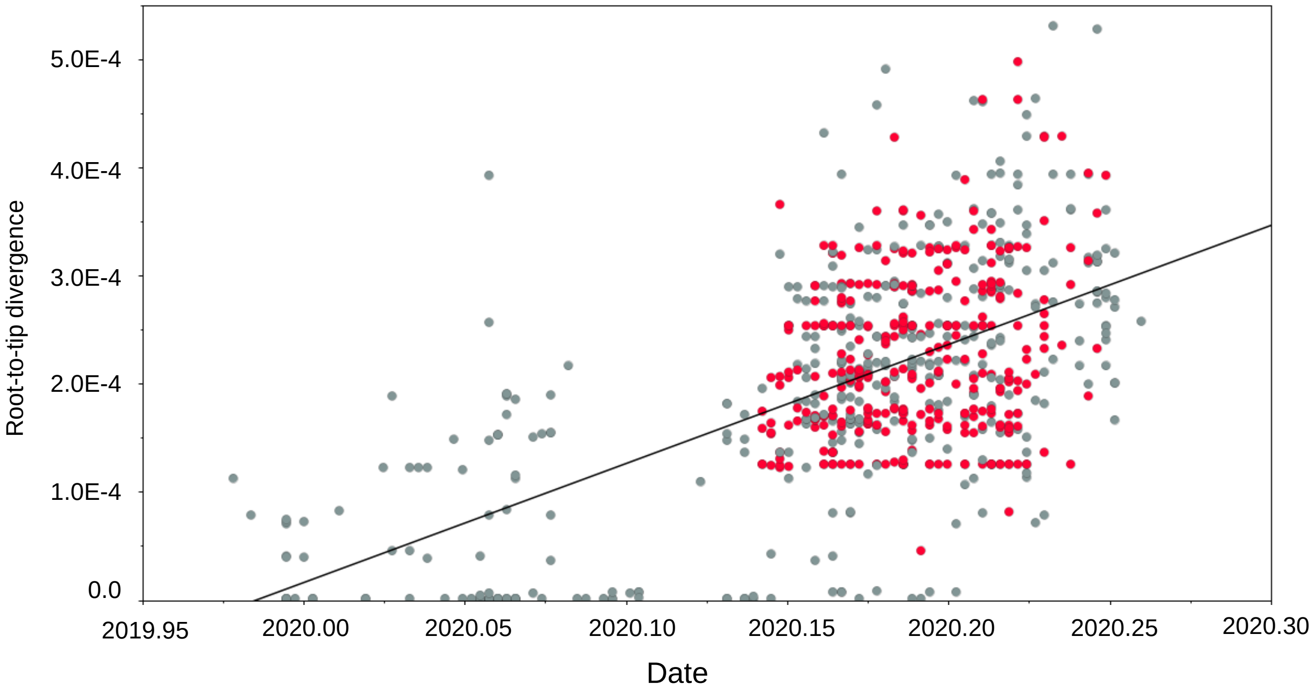

Supplementary Fig. 3. **Root-to-Tip Genetic Distance for SARS-CoV-2 Sequences in the Maximum Likelihood Tree Plotted against collection date.** The Pearson correlation coefficient between root-to-tip distance and collection date is 0.585. Sequences are colored by sampling location (Lombardy= red, other location = gray).

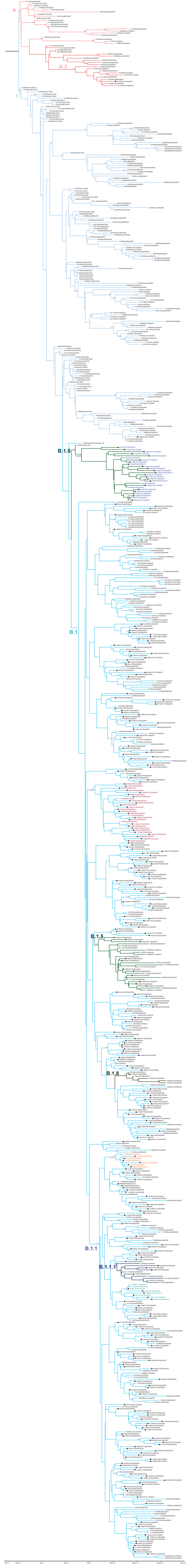

Supplementary Fig. 4. **Maximum clade credibility tree of SARS-CoV-2 genomes from Lombardy (taxa with red dots) and genomes from China and other countries (taxa without dots), according with lineages.** Lineages A and A.2 were defined by light and dark pink branches, B by light cyan branches, B.1 by light blue branches, B.1.1 by blue branches, B.1.5 and B.1.8 by light and dark green branches, B.1.1.1 by dark blue branches. Posterior probabilities >0.50 were shown at the corresponding nodes. Tips of sequences involved in local clusters supported by a posterior probability ≥0.98 were highlighted in red (A), in blue (B), in cyan (C), and in orange (D). Gisaid sequences come from Italy (n=15), East Europe (n=15), North Europe (n=10), South America (n=13), Africa (n=8) Japan (n=10), Oceania (n=25), West Asia (n=17), South Asia (n=14), Central Europe (n=30), East Asia (n=46), The Netherlands (n=41), South East Asia (n=19), North America (n=52), British Countries (n=47), China (n=55). Four independent chains were run for 50 million states.

Supplementary References

1. Crispell, J., Balaz, D. & Gordon, S.V. HomoplasyFinder: a simple tool to identify homoplasies on a phylogeny. *Microb. Genom.* **5**, e000245 (2019).
2. Isabel, S., *et al*. Evolutionary and structural analyses of SARS-CoV-2 D614G spike protein mutation now documented worldwide. *Sci. Rep*. **10**,14031 (2020).
3. Nguyen, L.T, Schmidt, H.A., von Haeseler, A., & Minh, B.Q. IQ-TREE: A fast and effective stochastic algorithm for estimating maximum likelihood phylogenies. *Mol. Biol. Evol*. **32**, 268-274 (2015).
4. Tavaré S. Some Probabilistic and Statistical Problems in the Analysis of DNA Sequences. *Lectures Math. Life Sci.* **17**, 57–86 (1986).
5. Rambaut, A., Lam, T. T., Max Carvalho, L. & Pybus, O. G. Exploring the temporal structure of heterochronous sequences using TempEst (formerly Path-O-Gen). *Virus evolution* **2**, vew007, (2016).
6. Suchard, M. A. *et al.* Bayesian phylogenetic and phylodynamic data integration using BEAST 1.10. *Virus evolution* **4**, vey016 (2018).
7. Ferreira, M.A.& Suchard, M.A. Bayesian analysis of elapsed times in continuous-time Markov chains. *Can. J. Stat.* **36**, 355–368 (2008).
8. Lemey, P., *et al*. Accommodating individual travel history, global mobility, and unsampled diversity in phylogeography: a SARS-CoV-2 case study. Preprint at https://www.biorxiv.org/content/10.1101/2020.06.22.165464v1 (2020).
9. Available at http://tree.bio.ed.ac.uk/software/figtree/.
