# Supplementary material for: Genomic epidemiology of SARS-CoV-2 reveals multiple lineages and early spread of SARS-CoV-2 infections in Lombardy, Italy
Source: Nat Commun. 2021 Jan 19;12:434. doi: 10.1038/s41467-020-20688-x (PMC7815831; doi:10.1038/s41467-020-20688-x)
Supplement: Supplementary file 3 — Descriptions of Additional Supplementary Files [file 41467_2020_20688_MOESM3_ESM.docx]

**Description of Additional Supplementary Files**

**Supplementary Data 1**

**Description:** The list of the whole-genome SARS-CoV-2 sequences (n=3244) retrieved from GISAID (gisaid.org) on 3 May 2020 and their corresponding accession numbers and lineages.

**Supplementary Data 2**

**Description:** The list of the 395 selected GISAID SARS-CoV-2 sequences considered for phylogenetic analyses and their corresponding accession numbers and lineages.

**Supplementary Data 3**

**Description:** The list of the 346 SARS-CoV-2 sequences obtained in this study and their corresponding ENA and GISAID accession numbers and lineages.

**Supplementary Data 4**

**Description:** The list of the significant homoplastic positions identified by HomoplasyFinder and TreeTime.
